# Walking and Walkability in Pre-Set and Self-Defined Neighborhoods: A Mental Mapping Study in Older Adults

**DOI:** 10.3390/ijerph15071363

**Published:** 2018-06-28

**Authors:** Malte Bödeker

**Affiliations:** 1 Department of Prevention and Health Promotion, School of Public Health, Bielefeld University, Post Office Box 10 01 31, Bielefeld D-33501, Germany; 2 Bavarian Health and Food Safety Authority, Institute of Public Health, Schweinauer Hauptstraße 80, Nuremberg D-90441, Germany; malte.boedeker@lgl.bayern.de; Tel.: +49-9131-6808-2944

**Keywords:** older adults, physical activity, walking, built environment, walkability, neighborhood, exposure area

## Abstract

Neighborhood walkability contributes to older adults’ walking. However, associations vary depending on the neighborhood definition applied as well as between objective and perceived walkability measures. Therefore, this study aimed to comparatively assess walkability indices for commonly used pedestrian network buffers and perceived neighborhood areas. A total of 97 adults aged ≥65 years answered a written physical activity questionnaire and 69 respondents participated in face-to-face interviews that involved mental mapping, i.e., to draw perceived neighborhood delineations on paper maps. Hierarchical regression analyses were used to compare the contribution of walkability indices for pre-set buffers and self-defined neighborhoods to older adults’ walking after adjusting for covariates. Results show that older adults’ self-defined neighborhoods are significantly larger, less home-centered, and more walkable than commonly used buffers. Furthermore, the variance accounted for in neighborhood walking increased from 35.9% to 40.4% (ΔR^2^ = 0.046; *p* = 0.029), when the walkability index was calculated for self-defined neighborhoods rather than pre-set buffers. Therefore, the study supports that geometric differences between pre-set buffers and older adults’ spatial ideas of perceived neighborhoods have a significant influence on estimated walkability effects and that exposure areas should be matched with the spatial dimension of outcome variables in future research.

## 1. Introduction

Regular physical activity is key for healthy ageing [1,2]. It reduces the risk of coronary heart disease, some cancers, type 2 diabetes, depression, cognitive impairment, and social isolation [3,4]. Yet, older adults (65 years or older) worldwide are often inactive so that identifying modifiable factors with a high level of reach and long-term impact has become a public health priority [4,5,6].

Walking substantially contributes to daily energy expenditure, is among the most popular activities in older adults and has a low risk of adverse outcomes such as falls and injuries [7,8,9]. Notably, errands and shopping are the main reason to leave home, which in turn is predictive physical activity in older adults [10]. Therefore, walkable neighborhoods with ease of access to shops and services may help to promote active aging on a population basis [11,12].

Recent systematic reviews confirmed that cumulative neighborhood walkability indices and single walkability attributes including destination access, land use mix, and residential density are associated to older adults’ physical activity and walking for transport in particular [13,14]. However, associations varied depending on the neighborhood definition applied as well as between objective and perceived walkability measures. Possible explanations include that neighborhood definitions of perceived measures (e.g., 10 to 15 min walk from home) correspond more closely to participants’ spatial ideas than those commonly used in Geographic Information Systems (GIS) like administrative districts or home-centered buffers [13,15,16].

Studies in young and middle-aged adults have examined residents’ spatial ideas using GIS and mental mapping, i.e., maps drawn by study participants to delineate perceived neighborhoods [17,18,19,20,21]. For example, Smith et al. [17] found that adults’ perceived neighborhoods shared an average (±standard deviation) of 16 ± 20% with one mile Euclidian buffers and 36 ± 47% with one mile pedestrian network buffers, respectively. Other research also found that environmental correlates of physical activity in older adults varied by neighborhood definition [22,23,24,25,26,27,28]. For example, Villanueva et al. reported that walking odds increased by 6% to 8% for a one-point change in neighborhood walkability indices that were based on 200 to 1600 m street network buffers [26]. However, GIS studies examining self-defined neighborhoods are scarce and walkability effects have not yet been assessed for perceived neighborhoods in older adults. Therefore, this study aimed to comparatively assess neighborhood walkability and its contribution to older adults’ walking for perceived neighborhoods and pre-set pedestrian network buffers.

## 2. Materials and Methods 

A cross-sectional study was carried out among community dwelling adults aged 65 years or older in Bielefeld, a historically evolved city with approximately 334,000 inhabitants in the north-west of Germany. The sampling frame consisted of senior housings estates listed by the local municipality (accessible housing and assisted living) and were located in the city center, in local district centers, and rural suburbs. A total of 97 residents from 26 senior housings estates answered written surveys and a subsample of 69 residents (71.1%) participated in mental mapping interviews. After exclusion of four participants, who had plotted walking destinations onto the maps but did not provide perceived neighborhood delineations, a total of 65 participants from 23 senior housing estates was included for analysis.

All subjects gave their informed consent for inclusion before they participated in the study. The study was conducted in accordance with the Declaration of Helsinki, and the protocol was approved by the Bielefeld School of Public Health Institutional Review Board.

### 2.1. Survey Measures

Surveys measures included self-reported physical activity, self-rated health, and sociodemographic characteristics. Habitual durations of neighborhood walking and total walking were defined as outcome measures in this study. Both outcomes were assessed with the Neighborhood Physical Activity Questionnaire (NPAQ; [29]) asking participants to recall a usual week and to report on walking durations within and outside the neighborhood, i.e., 10 to 15 walking minutes from home. The NPAQ has shown high test-retest reliability and moderate criterion-related validity against physical activity monitors in older adults [30,31]. Self-rated health was measured on a five-point scale asking participants “In general, would you say that your health is (1) excellent (2), very good, (3) good, (4) fair, or (5) poor?” [32]. Sociodemographic characteristics included age, gender, marital status, and socioeconomic status and were assessed using established items from the German Health Interview and Examination Survey for adults (Federal Health Reporting in Germany [33,34].

### 2.2. Mental Mapping Interviews

Perceived neighborhoods were assessed by mental mapping interviews following established procedures [17,21]. Face-to-face interviews were conducted by trained study assistants asking participants to draw perceived neighborhood delineations on paper maps. The maps originated from OpenStreetMap (OSM), displayed an area of 2 × 2 km, and included the place of residence in the center, local landmarks (e.g., urban parks, water bodies), and places of interest such as shops, public buildings, and public transport. The interviewers helped to orientate on the maps and explained that any size and shape including ‘circles, ovals, quads or pentagons’ were allowed. Subsequently, participants were asked to (a) recall and plot destinations they usually walk to and (b) free-hand draw what ‘you consider to be your neighborhood’ onto the maps. The reliability of mental map-based neighborhood delineations has been established elsewhere [21].

### 2.3. GIS Procedures

Geospatial data from the ‘Authoritative Real Estate Cadastre Information System’ (ALKIS) and OSM were processed in ArcGIS 10.3.1 (ESRI, Redlands, CA, USA). The ALKS data base contains administrative geographic information including utilization types of land parcel features and buildings. Utilization types were coded following the GIS procedures by Dobesova and Krivka [35] to allow for mixed use within the same building and land parcel feature, respectively. Street and path network data including access values for pedestrians originated from OSM; the accuracy of German OSM street and path network data has been evaluated elsewhere [36]. 

All GIS procedures were performed for perceived neighborhoods and home-centered pedestrian network buffers. Network buffers were computed based on the OSM pedestrian network and an impedance of 400 m, which is the most frequently used buffer size in older adults [13,14]. Perceived neighborhoods were digitized from paper maps that were scanned into GIS and georeferenced by assigning real-world coordinate reference points onto the images. 

Neighborhood walkability was assessed using the index developed by Frank et al. [37] with minor adaptions for the European context [35]. The composite score of four walkability attributes (household density, connectivity, land use mix, and retail floor area ratio) was calculated from standardized z-scores of these measures with a double weighting for connectivity [35,37]:

- *Household density* was defined by the number of households per square kilometer of residential area, i.e., residential and mixed-use land parcel features that included residence.
- *Connectivity* was assessed by the number of intersections (valence ≥ 3) per square kilometer in the OSM pedestrian network while merging all crossings within a radius of 15 m; water bodies were excluded from the reference area.
- *Land use mix* was calculated based on ALKIS land parcel features using Shannon’s index for the entropy of land use categories; water bodies were excluded from the analysis.
- The *retail floor area ratio* was defined by the share of commercial land parcel features covered by commercial buildings (footprints); commercial and mixed-use utilization types that included commerce were considered among buildings and land parcel features.

More detailed information on GIS procedures is available from Supplementary Materials.

### 2.4. Data Analysis

Descriptive statistics are presented as percentages or mean values and their standard deviations (±SD). Geometric comparisons between perceived neighborhoods and home-centered buffers included area (km^2^), overlap, and home-centeredness. Overlap was calculated by the percentage of shared area (intersect of both features) divided by the area of perceived neighborhoods. Home-centeredness was calculated by the Euclidian distance from polygon centroids to the senior housing estate location and expressed in meters.

All statistical tests were performed using the Complex Samples module in SPSS version 25 (IBM Corp., Armonk, NY, USA) to account for clustering in senior housing estates, sample weights to adjust for unequal probabilities of selection (Probability-Proportional-to-Size) and a *p*-value < 0.05 to indicate statistical significance. The Binomial test was used to compare the observed proportion of healthy older adults (reporting “good” to “excellent” self-rated health) to population-based estimates from the Federal Health Reporting System in Germany [34]. Unpaired *t*-test and χ^2^ tests were used for subsample comparisons. Paired *t*-tests were computed for individual-based mean differences in geometric measures and walkability attributes for perceived neighborhoods and home-centered buffers. Hierarchical regression analyses were used to compare the contribution of both walkability indices to older adults’ walking after adjusting for covariates. All independent variables had Variance Inflation Factor values < 10, indicating an acceptable level of multicollinearity, and the assumption of independence of errors was tested with the Durbin-Watson statistic. In the first step, age, gender, marital status, socioeconomic status, self-rated health, and buffer-based walkability were entered simultaneously to the regression models on neighborhood walking and total walking, respectively. Second, buffer-based walkability was removed and the walkability index for perceived neighborhoods was entered in lieu thereof. Partial *F*-tests were calculated to examine, if including the walkability index for perceived neighborhoods significantly improved the prediction of walking variables.

## 3. Results

The sample consisted of 65 older adults with a mean age (±SD) of 72.2 ± 8.6 years. Most participants were women (57.8%), lived with a partner (66.2%), and reported to be in good to excellent health (67.4%), which exceeds the population-based estimate of 51.5% for the age group ≥65 years (*p* < 0.001; [34]). Subsample comparisons showed that mental mapping participants were more likely to live with a partner than those denying interviews (*p* < 0.05). Age, gender, socioeconomic status and self-rated health were not significantly different between participants and dropouts. However, the proportion of participants reporting good to excellent health (67.4%)

### 3.1. Descriptive Results

Descriptive statistics are presented in Table 1. The mean of self-reported total walking was 68.5 ± 54.3 min per day with 68.5 ± 27.6% being accumulated by neighborhood walking. Comparing perceived neighborhoods and home-centered buffers, statistical significant mean differences were found for area, home-centeredness, the walkability index, and all its components (*p* < 0.05). Perceived neighborhoods covered a mean area of 0.76 ± 0.58 square kilometers and shared 10.1% to 100.0% with home-centered buffers (mean: 0.22 ± 0.59 km^2^); the average overlap was 35.8 ± 17.9%. The centroids of perceived neighborhoods located 194.6 ± 122.1 meters form senior housing estate locations, while the centroids of home-centered buffers were situated closer to the estates (mean: 32.8 ± 30.6 m). The walkability index varied −6.2 to 15.4 for perceived neighborhoods (mean: 1.00 ± 3.7 points) and −5.0 to 8.5 points for home-centered buffers (mean: −0.2 ± 3.7 points). Perceived neighborhoods were also characterized by lower household density, lower pedestrian connectivity, higher land use diversity, and higher retail floor area ratio than home-centered buffers.

### 3.2. Illustration of Neighborhood Geometries 

**Figure 1:**
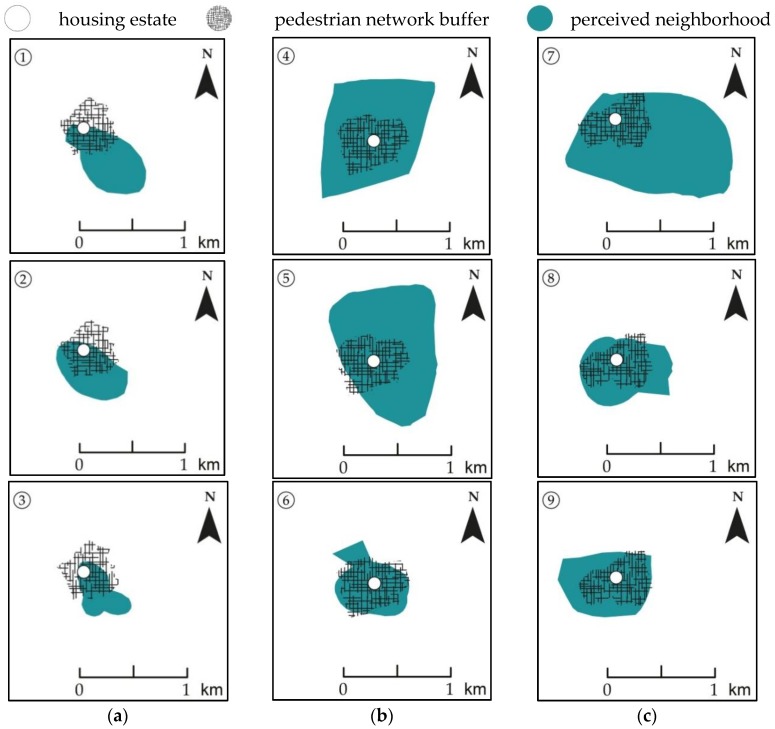
Perceived neighborhoods and home-centered pedestrian network buffers for (①–⑨) participants from (**a**–**c**) different senior housing estates.

Figure 1 illustrates the geometries of perceived neighborhoods and home-centered buffers. Spatial variability was found between different senior housing estates as well as between individuals living at the same address. For most participants, perceived neighborhoods largely covered home-centered buffers (Figure 1: ④–⑨) and many also exceeded them in one or more directions (Figure 1: ④, ⑤, ⑦). For others, there was a partial overlap between perceived neighborhoods and home-centered buffers (Figure 1: ①–③) and some also appeared to be home-centered like the buffers (Figure 1: ③, ⑥, ⑨).

### 3.3. Multivariate Prediction of Walking

Table 2 shows the multiple hierarchical regressions on neighborhood walking and total walking. Entering sociodemographic characteristics, self-rated health and buffer-based walkability in the first step, 35.9% of the variance in neighborhood walking was explained (R^2^ = 0.359; *p* < 0.001). Buffer-based walkability as well as self-rated health were identified as significant predictors. A one-point change in buffer-based walkability was associated with an estimated increase of 4.11 min per day (95%-CI: 1.65–6.57 min/day). Entering the walkability index for perceived neighborhoods instead of the buffer-based estimate in the second step, the variance accounted for in neighborhood walking statistical significantly increased to 40.4% (ΔR^2^ = 0.046; *p* = 0.029). A one-point change in the walkability index for perceived neighborhoods was associated with an estimated increase of 4.79 min per day (95%-CI: 2.50–7.08 min/day).

For the prediction of total walking, the model including sociodemographic characteristics, self-rated health and buffer-based walkability explained 42.5% of variance (R^2^ = 0.425; *p* < 0.001). Again, buffer-based walkability and self-rated health were significant predictors. A one-point change in buffer-based walkability was associated with an estimated increase of 7.58 min per day (95%-CI: 4.61–10.55 min/day) in total walking. After buffer-based walkability was replaced by the walkability index for perceived neighborhoods, 41.5% of the variance in total walking was accounted for (R^2^ = 0.415; *p* < 0.001). However, the change in R^2^ was not statistical significant for total walking (ΔR^2^ = −0.010; *p* = 0.320).

## 4. Discussion

This study examined GIS-based neighborhood walkability indices and their contribution in explaining older adults’ walking for pre-set 400 m pedestrian network buffers and perceived neighborhood areas. Findings showed that total walking and neighborhood walking related to neighborhood walkability, irrespective of neighborhood definition. This is in line with previous studies on total walking and neighborhood walkability [26,38,39,40] as well as on neighborhood walking and single neighborhood walkability attributes in older adults [27,41,42,43] and supports that pedestrian-friendly urban planning policies might help to promote active aging. 

However, despite relationships being found, this study also suggests that neighborhood definitions affect associations between walkability indices and walking. Based on the walkability index for perceived neighborhoods rather than pre-set 400 m pedestrian network buffers, an additional 4.6% of the variance in neighborhood walking was explained and the estimated increase for a one-point change in the walkability index enhanced from 4.11 to 4.79 min per day. This adds to previous findings that neighborhood walkability attributes and their associations to older adults’ physical activity vary by neighborhood definition [22,23,24,25,26,28]. Nevertheless, however, another finding of this study was that similar amounts of variance in total walking were explained by models including the walkability index for either home-centered buffers or perceived neighborhoods. This suggests that merely strengthening the context specificity of exposure measures does not improve the predictive power of built environment correlates. Nevertheless, the study supports previous calls to match exposure and outcome definitions spatially, i.e., to strengthen the context specificity of exposure and outcome measures at the same time [15,44,45,46]. 

Prospective assessments and spatiotemporal analyses of environmental exposures and health outcomes are required to examine how exposure area definitions affect associations and which areas are causally relevant for physical activity (Uncertain Geographic Context Problem; [47,48,49]). However, although causality cannot be inferred, mental mapping interviews and other qualitative methods provide valuable insights into areas that are relevant from the participants’ point of view and, therefore, might help to achieve greater precision in neighborhood studies [17,50]. Thus, it should be emphasized that older adults’ perceived neighborhoods were significantly larger, less home-centered, and that they shared little more than a third of the area with pre-set 400 m pedestrian network buffers. This is comparable to findings that administrative districts [19,20] and home-centered buffers [17,18,19,51] differ geometrically from perceived neighborhoods in other age groups and suggests that pre-set neighborhood definitions do not correspond accurately to participants’ spatial ideas. Furthermore, other mental mapping studies found that perceived neighborhoods have non-circular shapes [18,19,21], which is similar to the low level of home-centeredness in this study and suggests that participants include and exclude specific places, when being asked to delineate perceived neighborhoods.

Specifically, this study found that participants referred to more walkable areas and that they may have thought of shopping and mixed-use areas, since perceived neighborhoods were characterized by more diverse land use and higher retail floor area ratio than home-centered buffers. This adds to previous studies that have employed location monitoring to examine neighborhood walkability impacts on physical activity and activity space, i.e., a measure of spatial behavior generated form Global Positioning System data [48]. These studies consistently found that activity space differed geometrically from commonly used buffers around homes [52,53,54,55] and some also indicated that neighborhood walkability related to areas around homes that were actually utilized for physical activity [50,54,55,56]. Therefore, and consistent with the results from the present study, it may be suggested that commonly used buffers might underestimate neighborhood walkability and its contribution to physical activity in older adults. However, more research is needed to identify appropriate scales and zonings as well as neighborhood characteristics that impact on participants’ spatial ideas and perceived neighborhood delineations. Particularly, further study is warranted to compare perceived neighborhoods and activity spaces or integrate both approaches to establish exposure definitions that capture areas that are actually utilized as well as those being otherwise important from the residents’ perspective [50].

Finally, there are some strengths and limitations to keep in mind when interpreting these findings. This study is among the first to examine associations between older adults’ walking and neighborhood walkability indices for perceived neighborhoods and home-centered 400 m pedestrian network buffers. A major strength was the assessment of perceived neighborhoods to examine residents’ spatial ideas in mental mapping interviews [13,16]. The interviews were conducted by trained study assistants and with reference to established procedures [17,21]. As a result, 65 in 69 participants provided neighborhood delineations that were suitable for GIS analysis. However, personal contact may also be discussed as a reason for reduced study attendance [57], since a total of 28 (28.9%) participants refused mental mapping interviews, and singles were more likely to opt out than those living in domestic partnerships or married. Nevertheless, including a total of 65 participants from 23 different senior housing estates, this study allowed for individual and location-based comparisons of older adults’ perceived neighborhoods. Further strengths include the application of administrative geospatial data that allowed to calculate the retail floor area ratio, which has frequently been neglected in previous studies [58]. In addition, OSM data allowed to evaluate connectivity based on pedestrian rather than street networks [58,59], and it has been shown that OSM data provide more comprehensive walking route information than commercial databases [36]. 

Study limitations include the cross-sectional study design, recruitment of a predominantly female and healthy convenience sample, and reliance on self-reported physical activity. Cross-sectional studies demonstrate association rather than causation and might involve the risk of residential self-selection. However, studies adjusting for residential preferences indicate that neighborhood walkability is correlated with physical activity beyond residential self-selection [13]. Furthermore, others have reported under-reporting of physical activity among women and older adults [60,61,62], so that recall bias cannot be ruled out for this study. However, a validated physical activity questionnaire was used [30,31] and there is no reason to assume that response bias would affect the associations to buffer-based and perceived neighborhood-based walkability indices differently. Finally, it should be noted that the study accounted for areal clustering using complex sample statistics but did not employ multilevel analysis due to small numbers of participants per senior housing estate and corresponding model insufficiencies. Therefore, replication in larger and more diverse samples is warranted.

## 5. Conclusions

Examining exposure area definitions for neighborhood walkability, this study found a lack of correspondence between commonly used buffers and older adults’ spatial ideas of perceived neighborhood areas. Findings suggest that geometric differences between exposure areas affect the association between neighborhood walkability and neighborhood walking. This supports that findings may not be directly comparable between studies with different exposure area definitions and that exposure areas should be matched, i.e., to correspond spatially, with the spatial dimension of outcome variables. Exposure area definitions, therefore, merit particular consideration, at least when examining associations to location-based physical activity measures like neighborhood walking. Moreover, a lack of correspondence between commonly used buffers and perceived neighborhood areas also provides a possible explanation for inconsistencies in the literature on GIS-based vs. self-reported walkability measures and their associations to older adults’ physical activity. However, further studies in larger and more diverse samples are required to identify neighborhood characteristics that impact on self-defined neighborhood delineations and develop exposure area definitions that integrate participants’ spatial ideas and actual spatial behavior. In particular, spatiotemporal analyses are recommended to assess changes in physical activity, spatial behavior, and corresponding exposure areas, e.g., while residents explore neighborhoods after relocation. 

## Figures and Tables

**Table 1:** Descriptive statistics.

Variables	Mean	Standard Deviation	*p*-Value ^a^
**Walking (min/day)**			
Neighborhood Walking	46.20	32.12	n. a.
Total Walking	68.54	54.27	n. a.
**Area (km^2^)**			
Home-centered Buffer	0.22	0.59	<0.001
Perceived Neighborhood	0.76	0.58	
Overlap (%) ^b^	35.83	17.92	n. a.
**Home-Centeredness (m)** ^c^			
Home-centered Buffer	32.77	30.62	<0.001
Perceived Neighborhood	194.59	122.14	
**Walkability Index (point score)**			
Home-centered Buffer	−0.19	3.65	<0.001
Perceived Neighborhood	1.00	3.67	
**Household Density (units/km^2^)**			
Home-centered Buffer	6.105	3.259	0.025
Perceived Neighborhood	5.786	2.995	
**Pedestrian Connectivity (units/km^2^)**			
Home-centered Buffer	240.96	159.57	<0.001
Perceived Neighborhood	119.07	133.53	
**Land Use Mix (Shannon’s Index)**			
Home-centered Buffer	0.72	0.11	<0.001
Perceived Neighborhood	0.76	0.10	
**Retail Floor Area Ratio**			
Home-centered Buffer	0.27	0.13	<0.001
Perceived Neighborhood	0.31	0.13	

Complex sample statistics based on sample weights, *n* = 65 (*n_weighted_* = 58). ^a^ based on paired *t*-tests; ^b^ intersect of network buffer and perceived neighborhood (km^2^) by perceived neighborhood (km^2^); ^c^ Euclidian distance from polygon centroids to senior housing estate locations.

**Table 2:** Buffer-based and perceived neighborhood-based regression models for walking.

Predictors	Buffer-Based Models			Perceived Neighborhood Models		
	ß	B	95%-CI	ß	B	95%-CI
**Neighborhood Walking (min/day)**						
Constant		3.55	−73.46–80.55		−19.58	−95.01–55.84
Age	−0.01	−0.05	−0.89–0.79	0.40	0.14	−0.68–0.97
Gender (female)	−0.09	−5.80	−18.90–7.29	−0.07	−4.40	−17.03–8.23
Marital Status ^a^	−0.09	−6.36	−22.82–10.11	−0.05	−3.54	−18.91–11.84
Socioeconomic Status	−0.13	−1.73	−4.68–1.22	−0.07	−0.96	−3.83–1.91
Self-rated Health ^b^	0.49	**32.29**	**19.15–45.44**	0.42	**27.99**	**15.12–40.87**
Walkability Index	0.34	**4.11**	**1.65–6.57**	0.42	**4.79**	**2.50–7.08**
*n*	61			61		
*n_weighted_*	54			54		
unadjusted R^2^	0.359			0.404		
Δ unadjusted R^2^				0.046		
adjusted R^2^	0.304			0.354		
*F*-Statistic	**6.58; *p* < 0.001**			**7.99; *p* < 0.001**		
partial *F*-Statistic				**5.53; *p* = 0.029**		
**Total Walking (min/day)**						
Constant		25.14	−68.76–119.04		−5.55	−101.77–90.68
Age	−0.09	−0.47	−1.38–0.44	−0.06	−0.31	−1.25–0.63
Gender (female)	0.01	0.85	−14.92–16.63	0.04	3.22	−12.66–19.10
Marital Status ^a^	−0.09	−8.31	−27.05–10.43	−0.01	−0.85	−18.96–17.25
Socioeconomic Status	−0.11	−1.91	−5.33–1.51	−0.04	−0.69	−4.19–2.80
Self-rated Health ^b^	0.46	**41.25**	**25.54–56.96**	0.38	**34.51**	**18.59–50.42**
Walkability Index	0.47	**7.58**	**4.61–10.55**	0.45	**7.16**	**4.25–10.07**
*n*	65			65		
*n_weighted_*	58			58		
unadjusted R^2^	0.425			0.415		
Δ unadjusted R^2^				−0.010		
adjusted R^2^	0.384			0.373		
*F*-Statistic	**10.36; *p* < 0.001**			**9.96; *p* < 0.001**		
partial *F*-Statistic				1.37; *p* = 0.320		

Complex sample statistics based on sample weights. **Bold:** statistically significant at *p* < 0.05; ^a^ married or domestic partnership (vs. single, separated/divorced, widowed); ^b^ good to excellent (vs. fair to very poor).

## Supplementary Materials

The following are available online at http://www.mdpi.com/1660-4601/15/7/1363/s1, GIS Procedures.
