# Supplementary material for: Walking and Walkability in Pre-Set and Self-Defined Neighborhoods: A Mental Mapping Study in Older Adults
Source: Int J Environ Res Public Health. 2018 Jun 28;15(7):1363. doi: 10.3390/ijerph15071363 (PMC6068775; doi:10.3390/ijerph15071363)
Supplement: Supplementary file 1 [file ijerph-15-01363-s001.pdf]

Supplementary Materials: GIS Procedures

Symbols

$\boldsymbol{\rho}$	density
$\boldsymbol{p}$	area
t	intersection
b	building floor area footprint

Utilization types of a particular land parcel feature or building

Main categories (codes)

L	living
C	commercial
S	services
I	industrial
T	institutional
R	recreational
O	other (e.g. road space, agricultural area)
W	water (excluded)

Sub-categories (codes)
To allow for mixed land use within a particular land parcel feature or building, e.g. "L-C-S" to code a combined use of living, commercial, and services. Sub-categories formed discrete utilization types and were entered equally into the calculation of indices.

Equations

Pedestrian connectivity of a particular district

$$\boldsymbol{\rho_t} = \frac{\boldsymbol{t}}{\boldsymbol{p}} \tag{1}$$

where

$\boldsymbol{\rho_t}$	intersection density
$\boldsymbol{t}$	number of intersections (valence ≥3) based on the street, pedestrian, and bicycle networks, while merging all crossings within a straight line radius of 15 meters
$\boldsymbol{p}$	total land area of a district; water areas excluded

Household density of a particular district

$$\boldsymbol{\rho_h} = \frac{\boldsymbol{h}}{\boldsymbol{p_l}} \tag{2}$$

where:

$\boldsymbol{\rho_h}$	household density
$\boldsymbol{h}$	number of households
$\boldsymbol{p_l}$	area used for living, i.e. land parcel feature coded as living (L) or a sub-category including living (L-C, L-C-S, …)

Retail floor area ratio of a particular district

$$\boldsymbol{rFAR} = \frac{\sum \boldsymbol{b_c}}{\boldsymbol{p_c}} \tag{3}$$

where

$\boldsymbol{rFAR}$	retail floor area ratio
$\boldsymbol{b_c}$	floor area footprint of a particular commercial building, i.e. buildings coded as commercial (C) or a sub-category including commerce (L-C, L-C-S, …)
$\boldsymbol{p_c}$	area used for commerce, i.e. land parcel feature coded as commercial (C) or a sub-category including commerce (L-C, L-C-S, …)

Land use mix of a particular district

$$\boldsymbol{H}(\boldsymbol{S}) = \frac{-\sum_{i=1}^{k}[(\boldsymbol{p}_i) \times (\ln \boldsymbol{p}_i)]}{\ln \boldsymbol{k}} \qquad (4)$$

where

$\boldsymbol{H}(\boldsymbol{S})$ Entropy index (Shannon´s index)

$\boldsymbol{p_i}$ area of a particular utilization type (main and sub-categories) over the total land area of a district; water areas excluded.

$\boldsymbol{k}$ number of applicable utilization types in the particular district

Adapted from: Dobesova, Z.; Krivka, T. Walkability Index in the Urban Planning: A Case Study in Olomouc City. In *Advances in Spatial Planning*; Burian, J., Ed.: InTech: Shanghai, China, 2012, pp. 180–196.
